# Supplementary material for: Predictors of Symptom-Specific Treatment Response to Dietary Interventions in Irritable Bowel Syndrome
Source: Nutrients. 2022 Jan 17;14(2):397. doi: 10.3390/nu14020397 (PMC8780869; doi:10.3390/nu14020397)
Supplement: Supplementary file 1 [file nutrients-14-00397-s001.zip › Table S1.pdf]

Table S1. Mixed linear random-effect models including the effect of time, and all GSRS-IBS subscales as outcome*

	**Pain**			**Constipation**			**Diarrhea**			**Bloating**		
	β	*SE*	*p-value*	*β*	*SE*	*p-value*	*β*	*SE*	*p-value*	*β*	*SE*	*p-value*
(intercept)	3.01	0.12	**<0.0001**	1.48	0.11	**<0.0001**	1.33	0.05	**<0.0001**	3.73	0.15	**<0.0001**
Time	-0.17	0.03	**<0.0001**	-0.03	0.02	0.15	-0.06	0.01	**<0.0001**	-0.30	0.04	**<0.0001**

**NOTE: Gastrointestinal symptom rating scale for IBS, GSRS-IBS; effect size, β;standard error, SE; the GSRS-IBS subscales used in the linear mixed (i.e. random-effect) models were BoxCox transformed.* *The GSRS-IBS subscales used in the linear mixed (i.e. random-effect) models were BoxCox transformed*
