# Supplementary material for: Predictors of Symptom-Specific Treatment Response to Dietary Interventions in Irritable Bowel Syndrome
Source: Nutrients. 2022 Jan 17;14(2):397. doi: 10.3390/nu14020397 (PMC8780869; doi:10.3390/nu14020397)
Supplement: Supplementary file 1 [file nutrients-14-00397-s001.zip › Table S2.pdf]

**Table S2.** Mixed linear random-effect models including the effect of time and the diet factor, and all GSRS-IBS subscales as outcome*

| | Pain | | | Constipation | | | Diarrhea | | | Bloating | | |
|---|---|---|---|---|---|---|---|---|---|---|---|---|
| | *β* | *SE* | *p-value* | *β* | *SE* | *p-value* | *β* | *SE* | *p-value* | *β* | *SE* | *p-value* |
| (intercept) | 3.12 | 0.16 | **<0.0001** | 1.64 | 0.15 | **<0.0001** | 1.33 | 0.07 | **<0.001** | 3.86 | 0.21 | **<0.0001** |
| Time | -0.17 | 0.04 | **0.0004** | -0.05 | 0.03 | 0.18 | -0.06 | 0.02 | **0.003** | -0.26 | 0.05 | **<0.0001** |
| Diet (traditional IBS vs. low FODMAP) | -0.22 | 0.23 | 0.34 | -0.32 | 0.21 | 0.13 | -0.007 | 0.11 | 0.95 | -0.26 | 0.30 | 0.38 |
| Time*diet (traditional IBS vs. low FODMAP) | -0.02 | 0.06 | 0.80 | 0.02 | 0.05 | 0.64 | -0.001 | 0.03 | 0.96 | -0.07 | 0.07 | 0.35 |

**NOTE: Gastrointestinal symptom rating scale for IBS, GSRS-IBS; effect size, β;standard error, SE;* the GSRS-IBS subscales used in the linear mixed (i.e. random-effect) models were BoxCox transformed
