# Supplementary material for: Predictors of Symptom-Specific Treatment Response to Dietary Interventions in Irritable Bowel Syndrome
Source: Nutrients. 2022 Jan 17;14(2):397. doi: 10.3390/nu14020397 (PMC8780869; doi:10.3390/nu14020397)
Supplement: Supplementary file 1 [file nutrients-14-00397-s001.zip › Table S3.pdf]

Table S3. Mixed linear random-effect models including the effect of time, the diet factor, all different baseline factors, and all GSRS-IBS subscales as outcome*

	**Pain**			**Constipation**			**Diarrhea**			**Bloating**		
Independent variables: PHQ-12 and time	*β*	*SE*	*p-value*	*β*	*SE*	*p-value*	*β*	*SE*	*p-value*	*β*	*SE*	*p-value*
(intercept)	3.15	0.15	**<0.0001**	1.64	0.15	**<0.0001**	1.34	0.07	**<0.0001**	3.89	0.21	**<0.0001**
Time	-0.17	0.04	**0.001**	-0.04	0.03	0.21	-0.06	0.02	**0.004**	-0.26	0.05	**<0.0001**
Diet (low FODMAP)	-0.28	0.22	0.20	-0.32	0.22	0.14	-0.06	0.10	0.59	-0.35	0.30	0.25
Time*diet (traditional IBS vs. low FODMAP)	-0.02	0.06	0.71	0.03	0.05	0.61	0.001	0.03	0.96	-0.08	0.07	0.28
PHQ-12	0.35	0.15	**0.02**	0.04	0.15	0.77	0.12	0.07	0.09	0.31	0.21	0.14
Time*PHQ-12	-0.01	0.05	0.84	0.03	0.03	0.46	-0.004	0.02	0.84	0.04	0.05	0.40
PHQ-12*diet (traditional IBS vs. low FODMAP)	0.07	0.22	0.75	-0.04	0.22	0.87	0.03	0.10	0.75	-0.11	0.30	0.71
Time*PHQ-12*diet (traditional IBS vs. low FODMAP)	-0.01	0.06	0.92	-0.03	0.05	0.56	-0.01	0.03	0.75	-0.05	0.07	0.48
Independent variables: HADS and time	*β*	*SE*	*p-value*	*β*	*SE*	*p-value*	*β*	*SE*	*p-value*	*β*	*SE*	*p-value*
(intercept)	3.12	0.17	**<0.0001**	1.60	0.15	**<0.0001**	1.34	0.08	**<0.0001**	3.81	0.21	**<0.0001**
Time	-0.16	0.05	**0.001**	-0.04	0.03	0.22	-0.05	0.02	**0.01**	-0.24	0.05	**<0.0001**
Diet (traditional IBS vs. low FODMAP)	-0.25	0.24	0.30	-0.30	0.21	0.16	-0.02	0.11	0.85	-0.21	0.30	0.48
Time*diet (traditional IBS vs. low FODMAP)	-0.02	0.07	0.73	0.02	0.05	0.67	-0.003	0.03	0.91	-0.10	0.07	0.15
HADS	0.02	0.16	0.88	-0.18	0.14	0.21	0.03	0.07	0.64	-0.27	0.20	0.19
Time*HADS	0.01	0.04	0.84	0.01	0.03	0.68	0.01	0.02	0.51	0.11	0.05	**0.02**
HADS*diet (traditional IBS vs. low FODMAP)	0.07	0.24	0.76	0.26	0.22	0.22	0.0002	0.11	>0.99	0.30	0.31	0.33
Time*HADS*diet (traditional IBS vs. low FODMAP)	0.01	0.07	0.83	-0.02	0.05	0.71	-0.02	0.03	0.56	-0.05	0.07	0.54
Independent variables: VSI and time	*β*	*SE*	*p-value*	*β*	*SE*	*p-value*	*β*	*SE*	*p-value*	*β*	*SE*	*p-value*
(intercept)	3.11	0.16	**<0.0001**	1.64	0.14	**<0.0001**	1.33	0.07	**<0.0001**	3.87	0.21	**<0.0001**
Time	-0.17	0.04	**0.0004**	-0.05	0.03	0.19	-0.06	0.02	**0.004**	-0.27	0.05	**<0.0001**
Diet (traditional IBS vs. low FODMAP)	-0.17	0.22	0.45	-0.29	0.21	0.16	-0.01	0.10	0.90	-0.28	0.30	0.35
Time*diet (traditional IBS vs. low FODMAP)	-0.03	0.06	0.69	0.03	0.05	0.58	-0.001	0.03	0.96	-0.07	0.07	0.31
VSI	0.18	0.14	0.20	0.02	0.13	0.88	0.09	0.06	0.14	-0.08	0.18	0.67
Time*VSI	0.02	0.04	0.59	0.0002	0.03	>0.99	-0.0001	0.02	>0.99	0.09	0.04	**0.05**
VSI*diet (traditional IBS vs. low FODMAP)	0.31	0.24	0.19	0.42	0.22	0.06	0.14	0.11	0.20	0.52	0.32	0.10
Time*VSI*diet (traditional IBS vs. low FODMAP)	-0.02	0.07	0.77	-0.001	0.05	0.99	-0.02	0.03	0.47	-0.09	0.08	0.25
Independent variables: FODMAP intake and time	*β*	*SE*	*p-value*	*β*	*SE*	*p-value*	*β*	*SE*	*p-value*	*β*	*SE*	*p-value*
(intercept)	3.12	0.16	**<0.0001**	1.63	0.15	**<0.0001**	1.33	0.07	**<0.0001**	3.86	0.21	**<0.0001**
Time	-0.16	0.04	**0.0004**	-0.05	0.03	0.18	-0.06	0.02	**0.003**	-0.26	0.05	**<0.0001**

Diet (traditional IBS vs. low FODMAP)	-0.22	0.23	0.35	-0.32	0.21	0.13	-0.005	0.11	0.96	-0.27	0.30	0.37
Time*diet (traditional IBS vs. low FODMAP)	-0.02	0.06	0.80	0.02	0.05	0.64	-0.002	0.03	0.95	-0.07	0.07	0.35
FODMAP intake	-0.02	0.18	0.91	0.22	0.16	0.17	0.06	0.08	0.48	-0.19	0.23	0.40
Time*FODMAP intake	-0.06	0.05	0.21	-0.01	0.04	0.81	-0.03	0.02	0.19	-0.05	0.06	0.34
FODMAP intake*diet (traditional IBS vs. low FODMAP)	0.07	0.24	0.75	-0.07	0.21	0.72	-0.01	0.11	0.93	0.12	0.30	0.69
Time*FODMAP intake*diet (traditional IBS vs. low FODMAP)	0.12	0.06	0.06	0.03	0.05	0.51	0.04	0.26	0.16	0.10	0.07	0.20
Independent variables: Energy intake and time	*β*	*SE*	*p-value*	*β*	*SE*	*p-value*	*β*	*SE*	*p-value*	*β*	*SE*	*p-value*
(intercept)	3.11	0.16	**<0.0001**	1.64	0.15	**<0.0001**	1.33	0.07	**<0.0001**	3.86	0.20	**<0.0001**
Time	-0.17	0.04	**0.0003**	-0.05	0.03	0.18	-0.06	0.02	**0.003**	-0.26	0.05	**<0.0001**
Diet (traditional IBS vs. low FODMAP)	-0.22	0.23	0.34	-0.32	0.21	0.13	-0.01	0.11	0.95	-0.26	0.29	0.36
Time*diet (traditional IBS vs. low FODMAP)	-0.02	0.06	0.79	0.02	0.05	0.64	-0.001	0.03	0.96	-0.07	0.07	0.36
Energy intake	0.05	0.16	0.74	-0.01	0.15	0.94	-0.02	0.07	0.73	-0.29	0.20	0.15
Time*energy intake	-0.06	0.04	0.16	-0.001	0.03	0.97	-0.01	0.02	0.48	0.02	0.05	0.75
Energy intake*diet (traditional IBS vs. low FODMAP)	-0.33	0.23	0.15	0.06	0.21	0.78	-0.07	0.11	0.51	-0.10	0.29	0.72
Time*energy intake*diet (traditional IBS vs. low FODMAP)	-0.02	0.06	0.81	-0.02	0.05	0.71	-0.01	0.03	0.75	-0.01	0.07	0.88
Independent variables: Dysbiosis Index (DI) score (in tertiles) and time	*β*	*SE*	*p-value*	*β*	*SE*	*p-value*	*β*	*SE*	*p-value*	*β*	*SE*	*p-value*
(intercept)	2.81	0.33	**<0.0001**	1.53	0.30	**<0.0001**	1.33	0.15	**<0.0001**	3.98	0.43	**<0.0001**
Time	-0.14	0.09	0.12	0.07	0.07	0.27	-0.06	0.04	0.12	-0.23	0.10	**0.03**
Diet (traditional IBS vs. low FODMAP)	-0.03	0.51	0.95	-0.40	0.46	0.39	0.21	0.23	0.36	-0.62	0.65	0.35
Time*diet (traditional IBS vs. low FODMAP)	0.15	0.14	0.28	0.06	0.10	0.57	-0.02	0.06	0.68	-0.05	0.16	0.75
DI socre (lowest vs. highest tertile)	0.21	0.47	0.65	-0.30	0.43	0.49	-0.10	0.21	0.65	-0.85	0.60	0.16
DI score (mid vs. highest tertile)	0.48	0.40	0.23	0.34	0.37	0.35	0.05	0.18	0.76	0.15	0.51	0.77
DI score (lowest vs. mid tertile)	-0.27	0.40	0.51	-0.64	0.37	0.08	-0.15	0.18	0.40	-1.01	0.51	**0.05**
Time*DI score (lowest vs. highest tertile)	-0.05	0.13	0.70	-0.18	0.09	**0.05**	0.04	0.05	0.52	0.12	0.15	0.43
Time*DI score (mid vs. highest tertile)	-0.02	0.11	0.86	-0.15	0.08	0.07	-0.01	0.05	0.88	-0.11	0.13	0.40
Time*DI score (lowest vs. mid tertile)	-0.03	0.11	0.78	-0.04	0.08	0.63	0.04	0.05	0.36	0.22	0.13	0.08
DI score (lowest vs. highest tertile)*diet (traditional IBS vs. low FODMAP)	-0.39	0.66	0.56	0.48	0.60	0.43	-0.31	0.30	0.31	1.03	0.85	0.23

DI score (mid vs. highest tertile)*diet (traditional IBS vs. low FODMAP)	-0.06	0.61	0.93	-0.05	0.56	0.92	-0.18	0.28	0.51	0.24	0.78	0.76
DI score (lowest vs. mid tertile)*diet (traditional IBS vs. low FODMAP)	-0.33	0.54	0.54	0.53	0.49	0.28	-0.13	0.24	0.61	0.78	0.69	0.26
Time*DI score (lowest vs. highest tertile)*diet (traditional IBS vs. low FODMAP)	-0.25	0.18	0.17	-0.01	0.13	0.96	0.005	0.08	0.95	-0.27	0.21	0.20
Time*DI score (mid vs. highest tertile)*diet (traditional IBS vs. low FODMAP)	-0.15	0.17	0.37	-0.04	0.12	0.72	0.04	0.07	0.62	0.13	0.19	0.49
Time*DI score (lowest vs. mid tertile)*diet (traditional IBS vs. low FODMAP)	-0.10	0.15	0.49	0.04	0.11	0.73	-0.03	0.06	0.62	-0.40	0.17	**0.02**
Independent variables: Oligosaccharide intake and time	*β*	*SE*	*p-value*	*β*	*SE*	*p-value*	*β*	*SE*	*p-value*	*β*	*SE*	*p-value*
(intercept)	3.00	0.28	**<0.0001**	1.48	0.27	**<0.0001**	1.18	0.14	**<0.0001**	3.82	0.38	**<0.0001**
Time	-0.02	0.08	0.83	-0.05	0.06	0.42	-0.02	0.03	0.60	-0.16	0.09	0.09
Diet (traditional IBS vs. low FODMAP)	0.83	0.41	**0.04**	0.11	0.40	0.78	0.25	0.20	0.21	0.49	0.56	0.38
Time*diet (traditional IBS vs. low FODMAP)	-0.22	0.12	0.06	0.01	0.09	0.91	-0.05	0.05	0.32	-0.39	0.13	**0.004**
Oligosaccharide intake	0.09	0.18	0.64	0.12	0.18	0.49	0.12	0.09	0.17	0.03	0.25	0.90
Time*Oligosaccharide intake	-0.12	0.05	**0.03**	0.004	0.04	0.91	-0.03	0.02	0.18	-0.08	0.06	0.17
Oligosaccharide intake*diet (traditional IBS vs. low FODMAP)	-0.86	0.28	**0.002**	-0.35	0.28	0.21	-0.20	0.14	0.14	-0.61	0.38	0.11
Time*Oligosaccharide intake*diet (traditional IBS vs. low FODMAP)	0.16	0.08	**0.05**	0.01	0.06	0.88	0.04	0.03	0.27	0.26	0.09	**0.01**
Independent variables: Polyols intake and time	*β*	*SE*	*p-value*	*β*	*SE*	*p-value*	*β*	*SE*	*p-value*	*β*	*SE*	*p-value*
(intercept)	3.12	0.21	**<0.0001**	1.61	0.19	**<0.0001**	1.36	0.09	**<0.0001**	3.64	0.27	**<0.0001**
Time	-0.17	0.06	**0.004**	-0.07	0.04	0.10	-0.07	0.02	**0.005**	-0.22	0.07	**0.001**
Diet (traditional IBS vs. low FODMAP)	-0.11	0.29	0.70	-0.23	0.26	0.39	0.04	0.13	0.73	0.03	0.37	0.93
Time*diet (traditional IBS vs. low FODMAP)	-0.01	0.08	0.93	0.05	0.06	0.37	0.02	0.03	0.48	-0.08	0.09	0.37
Polyols intake	0.002	0.12	0.99	-0.02	0.11	0.82	0.02	0.05	0.65	-0.20	0.15	0.17
Time*Polyols intake	-0.004	0.03	0.89	-0.02	0.02	0.33	-0.01	0.01	0.39	0.04	0.04	0.30
Polyols intake*diet (traditional IBS vs. low FODMAP)	0.10	0.16	0.51	0.09	0.14	0.55	0.05	0.07	0.49	0.27	0.20	0.19
Time*Polyols intake*diet (traditional IBS vs. low FODMAP)	0.01	0.04	0.85	0.03	0.03	0.40	0.02	0.02	0.21	-0.01	0.05	0.82

Independent variables: Disaccharide intake and time	*β*	*SE*	*p-value*	*β*	*SE*	*p-value*	*β*	*SE*	*p-value*	*β*	*SE*	*p-value*
(intercept)	2.71	0.32	**<0.0001**	0.85	0.27	**0.003**	1.29	0.15	**<0.0001**	3.93	0.43	**<0.0001**
Time	-0.04	0.09	0.67	0.09	0.07	0.16	-0.02	0.04	0.66	-0.18	0.10	0.09
Diet (traditional IBS vs. low FODMAP)	-0.18	0.45	0.69	0.06	0.38	0.87	-0.04	0.21	0.85	-0.44	0.60	0.46
Time*diet (traditional IBS vs. low FODMAP)	-0.19	0.12	0.14	-0.16	0.09	0.09	-0.06	0.05	0.24	-0.20	0.15	0.18
Disaccharide intake	0.17	0.11	0.15	0.32	0.10	**0.001**	0.02	0.05	0.72	-0.03	0.15	0.85
Time*Disaccharide intake	-0.05	0.03	0.10	-0.06	0.02	**0.02**	-0.02	0.01	0.21	-0.03	0.04	0.36
Disaccharide intake*diet (traditional IBS vs. low FODMAP)	-0.02	0.16	0.92	-0.15	0.13	0.25	0.01	0.07	0.86	0.08	0.21	0.72
Time*Disaccharide intake*diet (traditional IBS vs. low FODMAP)	0.07	0.04	0.12	0.07	0.03	**0.02**	0.02	0.02	0.18	0.05	0.05	0.31
**Independent variables: Monosaccharide intake and time**	*β*	*SE*	*p-value*	*β*	*SE*	*p-value*	*β*	*SE*	*p-value*	*β*	*SE*	*p-value*
(intercept)	3.24	0.27	**<0.0001**	1.84	0.24	**<0.0001**	1.33	0.12	**<0.0001**	4.10	0.34	**<0.0001**
Time	-0.13	0.07	0.07	-0.08	0.06	0.13	-0.03	0.03	0.27	-0.21	0.08	**0.02**
Diet (traditional IBS vs. low FODMAP)	-0.40	0.39	0.30	-0.52	0.35	0.14	-0.01	0.18	0.96	-0.63	0.50	0.21
Time*diet (traditional IBS vs. low FODMAP)	-0.09	0.11	0.39	0.03	0.08	0.74	-0.03	0.04	0.50	-0.08	0.12	0.54
Monosaccharide intake	-0.10	0.15	0.53	-0.13	0.14	0.35	-0.0003	0.07	>0.99	-0.19	0.19	0.34
Time*Monosaccharide intake	-0.02	0.04	0.67	0.03	0.03	0.32	-0.01	0.02	0.42	-0.03	0.05	0.48
Monosaccharide intake*diet (traditional IBS vs. low FODMAP)	0.14	0.23	0.55	0.13	0.21	0.52	0.01	0.10	0.95	0.28	0.29	0.33
Time*Monosaccharide intake*diet (traditional IBS vs. low FODMAP)	0.05	0.06	0.43	-0.01	0.05	0.90	0.02	0.03	0.49	0.001	0.07	0.98

**NOTE: Gastrointestinal symptom rating scale for IBS, GSRS-IBS; patient health questionnaire-12, PHQ-12; hospital anxiety and depression scale, HADS; visceral sensitivity index, VSI; dysbiosis index, DI; fermentable oligo-, di-, monosaccharides, and polyols, FODMAP; effect size, β;standard error, SE; the GSRS-IBS subscales used in the linear mixed (i.e. random-effect) models were BoxCox transformed.*
